# Work and Activity Impairment in Individuals with Essential Tremor

**DOI:** 10.5334/tohm.1034

**Published:** 2025-07-02

**Authors:** Margaret E. Gerbasi, Rodger J. Elble, Holly A. Shill, Eddie Jones, Alexander Gillespie, John Jarvis, Elizabeth Chertavian, Zachary Smith, Ludy C. Shih

**Affiliations:** 1 Sage Therapeutics, Inc. Cambridge, MA, US; 2 Southern Illinois University School of Medicine, Springfield, IL, US; 3 Barrow Neurological Institute, Phoenix, AZ, US; 4 Adelphi Real World, Adelphi Mill, Bollington, UK; 5 Medicus Economics, Milton, MA, US; 6 Beth Israel Deaconess Medical Center, Boston, MA, US; 7 Harvard Medical School, Boston, MA, US

**Keywords:** essential tremor, care partner burden, activities of daily living

## Abstract

**Background:**

Essential tremor (ET) affects nearly 7 million people in the United States and consists of upper limb tremor that can affect activities of daily living, including activities related to work. Research examining the effect of ET on work productivity is limited and there are no studies using validated work productivity instruments.

**Methods:**

Clinic-based data were collected between March 2021 and August 2021 from US physicians participating in the Adelphi ET Disease Specific Programme (DSP). Patients were evaluated with the Essential Tremor Rating Assessment Scale (TETRAS), Quality of Life in Essential Tremor (QUEST) questionnaire, and the Work Productivity and Activity Impairment (WPAI) questionnaire. Statistical associations between tremor severity and work productivity were examined.

**Results:**

A total of 1,003 ET patients were identified, and 420 patients completed the WPAI questionnaire and were included in this study. Activity impairment was independently associated with tremor severity, adjusting for age, full-time vs. part-time employment, household income, depression, and anxiety. Of those who were employed (n = 165), 133 (80.6%) were employed full-time, and 141 (85.5%) had some level of work impairment. Work impairment was also independently associated with tremor severity, adjusting for the same covariates. Among those patients for whom QUEST responses were available, 47% (64/135) of patients with ET working full-time and 88% (36/41) of those working part-time reported that tremor interfered with work.

**Discussion:**

Work impairment is significantly correlated with tremor severity. Although a minority of patients in this clinic-based cohort were identified as employed, nearly all reported a negative impact on work performance.

## Introduction

Essential tremor (ET) is one of the most common movement disorders in the US, affecting an estimated 6.8 million US adults [1,2]. Primary manifestations of ET include action tremor in the upper limbs that can significantly impact physical activity [3] and activities of daily living (ADLs) [4,5,6,7]. However, research examining the effect of ET on work productivity is limited. One prior study showed that ET patients reported a negative impact on work, compared to age-matched adults without ET [8]. In addition, ET patients reported interference with their job or profession in two prior studies [4,9], though the two samples differed significantly. The study involving a much younger sample of ET patients reported a higher percentage (46% vs. 8.5% [4]) of patients reporting that their tremor “always” interfered with their job of profession [9]. Prevalence estimates reveal that a considerable number of ET patients are 69 years old or younger, comprising roughly 50% of those living with ET [10] and about 40% of diagnosed cases [11]. These data highlight the potential impact of ET on work productivity and a potential benefit of effective ET treatments. However, no data assessing work productivity in patients with ET using validated work productivity instruments have been reported.

The goals of this study were to describe activity impairment in a clinic-based cohort of patients with ET, to characterize the work productivity impact of ET among employed patients, including both absenteeism and presenteeism, and to examine the association between productivity and activity impairment and measures of ET tremor severity, using a previously validated work productivity instrument, the Work Productivity and Activity Impairment (WPAI) questionnaire [12].

## Methods

The study used clinic-based real-world data collected in the US from March 2021 to August 2021 through an Adelphi ET Disease Specific Programme (DSP)™ [13]. Physicians involved in the management of patients with ET in the US were identified from public lists of healthcare professionals and included in the study if they were currently practicing physicians who treated 10 or more ET patients in a typical month. Each eligible and consenting physician provided information on their next 10 consecutive patients with ET, regardless of the reason for the visit. Patients with ET were ≥18 years old and not currently involved in a clinical trial.

A total of 1,003 ET patients were identified, and 420 patients completed the Work Productivity and Activity Impairment (WPAI) questionnaire and comprised the Assessment Cohort. WPAI is a 6-item questionnaire that assesses impairment of paid work (absenteeism and presenteeism) and unpaid work and regular daily activities (e.g., household work, shopping, childcare, etc.) during the past seven days [12]. Activity impairment was based upon response to question 6 of the WPAI: “During the past seven days, how much did your health problems affect your ability to perform your normal daily activities, excluding your job?” on a 0–10 point scale, with 0 indicating no impairment and 10 indicating 100% impairment. Presenteeism was based on question 5: “During the past seven days, how much did your health problems affect your productivity while you were working?“ and was also scored 0–10. For analysis purposes, activity impairment and presenteeism scores were expressed as percentages (score × 10). Absenteeism was defined as the ratio of total hours missed to the total number of expected work hours, based on questions (Q) 2 and 4 (i.e., Q2/(Q2+Q4)). Overall work impairment was calculated as the sum of absenteeism plus the product of presenteeism and the time spent working (e.g., Q2/(Q2 + Q4) + [(1 – (Q2/(Q2 + Q4))) × (Q5/10)]. The “Employed Subset” in this study consisted of those patients (n = 165) who provided work hour information on WPAI questions 2, 4 and 5.

Tremor severity was assessed as a continuous measure with the Essential Tremor Rating Scale (TETRAS), a clinician-administered clinical rating scale with Performance (P) and activities of daily living (ADL) subscales [14]. TETRAS-P quantifies tremor of the head, face, voice, upper and lower limbs with 0–4 ratings for a total of 68 points. Item 4 of the TETRAS-P measures right and left upper limb action tremor in the forward-horizontal and wing postures and in the finger-nose-finger movement task, producing a maximum score of 24. The TETRAS-ADL is a clinician-patient interview in which the clinician and patient produce a 0–4 consensus rating of the impact of tremor on speech (one item), upper limb function (10 items), and social function (one item). Patient-reported impact of tremor on quality of life was assessed with the Quality of Life in Essential Tremor Questionnaire (QUEST) [15].

Demographics including age, sex, race/ethnicity, insurance coverage, medical co-morbidities, and patient-reported employment status (full-time, part-time, retired, unemployed, student) were collected for all ET patients in the dataset. Pearson correlations and ordinary least squares (OLS) or logit regression models were employed to test the association between absenteeism and tremor severity, presenteeism with tremor severity, and activity impairment with tremor severity, with core models including relevant covariates (age, employment status, household income, anxiety, and depression). Tremor severity was measured by TETRAS-ADL, TETRAS-P, and TETRAS-P item 4. Expanded models include core model covariates plus sex, educational level and Charlson Comorbidity Index (CCI) [16].

## Results

Table 1 presents the characteristics of the 2 cohorts analyzed in this study. Across the entire Assessment Cohort (n = 420), patients were a mean (SD) 64.4 (13.8) years old, and 47% were female. In the subset of patients who were employed, referred to as the Employed Subset (n = 165), patients were 55.0 years old on average [SD = 10.1], and 41% were female. Most were employed full-time (n = 133 [80.6%] vs part-time n = 32 [19.4%]). The top comorbidities in both cohorts were hypertension, anxiety, hyperlipidemia, and depression.

**Table 1:** Demographics and ET disease characteristics.


		ASSESSMENT COHORT (n = 420)		EMPLOYED SUBSET (n = 169)	

Sex, % (n)	Female	47%	(198)	41%	(70)

Age (years), mean (sd)	Age	64.4	(13.8)	55.0	(10.3)

BMI, mean (sd)	BMI	27.0	(4.7)	28.0	(4.9)

Age category, % (n)	18–64	45.7%	(192)	84.0%	(142)

	65+	54.3%	(228)	16.0%	(27)

Race/Ethnicity, % (n)	White/Caucasian	79.8%	(335)	82.8%	(140)

	Asian	2.1%	(9)	0.6%	(1)

	African American	10.5%	(44)	11.2%	(19)

	Hispanic	4.0%	(17)	2.4%	(4)

	Other	3.6%	(15)	3.0%	(5)

Insurance coverage, % (n)	Medicare	50.2%	(211)	11.2%	(19)

	Medicaid	3.1%	(13)	1.2%	(2)

	Commercial	43.6%	(183)	83.4%	(141)

	Other	3.1%	(13)	4.1%	(7)

Employment status, % (n)	Full time	36.7%	(154)	79.9%	(135)

	Part time	10.0%	(42)	20.1%	(34)

	Retired	42.4%	(178)	N/A	N/A

	Unemployed	10.5%	(44)	N/A	N/A

	Student	0.5%	(2)	N/A	N/A

Education level achieved, % (n)	Less than high school	5.2%	(22)	0.0%	(0)

	High school	23.8%	(100)	9.5%	(16)

	College degree (2-year bachelor)	36.9%	(155)	53.8%	(91)

	College degree (4-year bachelor)	18.8%	(79)	21.3%	(36)

	Graduate degree or higher	11.0%	(46)	11.8%	(20)

	Other	4.3%	(18)	3.6%	(6)

Household income	$50k or less	24.8%	(104)	16.0%	(27)

	$50k–$75k	15.2%	(64)	13.6%	(23)

	$75k–$100k	17.6%	(74)	26.0%	(44)

	$100k–$125k	6.7%	(28)	6.5%	(11)

	$125k–$150k	7.6%	(32)	7.1%	(12)

	$150k+	8.6%	(36)	13.0%	(22)

	Did not report	19.5%	(82)	17.8%	(30)

Current home circumstances, % (n)	Residing at a nursing home	1.9%	(8)	0.0%	(0)

	Residing with family	82.4%	(346)	89.3%	(151)

	Residing alone	13.6%	(57)	9.5%	(16)

	Other	1.0%	(4)	0.0%	(0)

	Unknown	1.2%	(5)	1.2%	(2)

Has someone responsible for daily needs, % (n)	Yes	31.7%	(133)	18.3%	(31)

	No	64.0%	(269)	78.1%	(132)

	Unknown	4.3%	(18)	3.6%	(6)

Employment limited due to ET, % (n)	Yes	2.4%	(10)	0.0%	(0)

Receiving disability income, % (n)	Yes	6.7%	(28)	1.2%	(2)

TETRAS scores, mean (sd)	TETRAS activities of daily living (ADL) score	17.4	(9.8)	15.1	(8.6)

	TETRAS ADL Composite score	8.6	(8.4)	6.2	(6.9)

	TETRAS Performance score	22.7	(11.8)	20.2	(11.1)

	TETRAS Performance Item 4 score	9.8	(4.8)	8.9	(4.3)

	TETRAS total score	40.1	(20.8)	35.3	(19.0)

QUEST scores, mean (sd)	QUEST total score	24.5	(19.4)	20.0	(16.3)

	QUEST communication	18.1	(24.2)	12.4	(19.8)

	QUEST work finances	14.1	(21.1)	13.2	(16.9)

	QUEST hobbies and leisure	25.9	(32.4)	19.7	(29.1)

	QUEST physical	36.8	(23.4)	30.4	(19.8)

	QUEST psychosocial	27.5	(21.9)	24.4	(17.7)

EQ-5D scores, mean (sd)	EQ-5D index score	0.74	(0.22)	0.82	(0.17)

	EQ-5D visual analogue scale (VAS) score	75.0	(15.2)	78.8	(13.9)

Start of ET symptoms, % (n)	Childhood	2.4%	(10)	2.4%	(4)

	20–29	4.0%	(17)	7.1%	(12)

	30–39	4.3%	(18)	5.9%	(10)

	40–49	14.0%	(59)	24.9%	(42)

	50–59	29.0%	(122)	42.6%	(72)

	60–69	28.6%	(120)	16.6%	(28)

	70–79	13.3%	(56)	0.6%	(1)

	80–89	3.1%	(13)	0.0%	(0)

	Unknown	1.2%	(5)	0.0%	(0)

ET treatment status/history, % (n)	Currently prescribed a drug for treating ET	83.1%	(349)	88.8%	(150)

	Previously prescribed a drug for treating ET	7.6%	(32)	3.0%	(5)

	Has never been prescribed ET treatment	9.3%	(39)	8.3%	(14)

Currently-prescribed ET treatments, % (n)	Propranolol	35.7%	(150)	37.3%	(63)

	Primidone	32.1%	(135)	31.4%	(53)

	Atenolol	10.5%	(44)	16.0%	(27)

	Sotalol	0.5%	(2)	0.0%	(0)

	Nadolol	2.6%	(11)	3.0%	(5)

	Alprazolam	7.6%	(32)	8.9%	(15)

	Clonazepam	5.5%	(23)	3.0%	(5)

	Lorazepam	3.6%	(15)	4.1%	(7)

	Diazepam	1.9%	(8)	2.4%	(4)

	Gabapentin	6.0%	(25)	1.2%	(2)

	Pregabalin	4.3%	(18)	4.1%	(7)

	Topiramate	8.1%	(34)	5.9%	(10)

	Zonisamide	0.0%	(0)	0.0%	(0)

	Clozapine	0.2%	(1)	0.0%	(0)

	Nimodipine	0.0%	(0)	0.0%	(0)

	Botulinum toxin	3.1%	(13)	2.4%	(4)

	Deep-brain stimulation (DBS)	0.7%	(3)	0.6%	(1)

	Cala Trio (wrist-worn device)	1.9%	(8)	3.6%	(6)

Past ET-related procedures, % (n)	Deep-brain stimulation (DBS)	7.9%	(33)	5.9%	(10)

	Thalamotomy	4.0%	(17)	3.0%	(5)

	Magnetic resonance-guided focused ultrasound (MRgFUS)	4.8%	(20)	5.3%	(9)

	Other	3.3%	(14)	4.1%	(7)

	None	77.1%	(324)	75.7%	(128)

	Unknown	11.2%	(47)	12.4%	(21)


### Activity Impairment and Association with Tremor Severity

**Figure 1:**
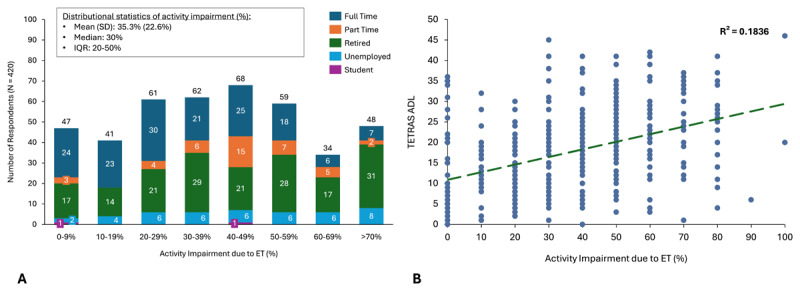
**Activity impairment in ET patients stratified by employment status and association with tremor severity in ET patients. A)** Number of respondents and levels of activity impairment, stratified by full-time, part-time, retired, unemployed, and student status. **B)** Activity impairment as measured by question 6 on the Work Productivity and Activity Impairment questionnaire. Tremor severity as measured by TETRAS-ADL, R² = 0.1836.

Most patients (373/420; 88.8%) reported some activity impairment. In the entire Assessment Cohort, the mean degree of activity impairment was 35.3% (SD = 22.6%) with rates of activity impairment varying across different types of work status (Figure 1A). The Employed Subset reported a similar level of activity impairment compared with the overall Assessment Cohort (mean activity impairment 29.0% [SD = 22.5%]). Activity impairment in the Assessment Cohort was significantly associated with worsening (increasing) TETRAS-ADL scores, TETRAS-P, and TETRAS-P item 4 scores (p < 0.001 for trend; Figure 1B). Higher patient activity impairment was moderately associated with greater tremor severity (rs = 0.37–0.43). The magnitude of relationships was not impacted by inclusion of covariates (age, full-time vs part-time, household income, depression, anxiety) in the core regression models. Sensitivity analyses included expanded models adjusting for core model covariates plus sex, education level, and Charlson Comorbidity Index (Table 2).

**Table 2:** Activity impairment and association with tremor severity as measured by TETRAS subscales.


MODEL STRUCTURE	TETRAS ADL SCORE		

OLS model	Bivariate^1^	Core^2^	Expanded Model^3^

	0.0099***	0.0082***	0.0079***

	**TETRAS PERFORMANCE SCORE**		

OLS model	Bivariate^1^	Core^2^	Expanded Model^3^

	0.0083***	0.0069***	0.0067***

	**TETRAS PERFORMANCE ITEM 4 SCORE**		

OLS model	Bivariate^1^	Core^2^	Expanded Model^3^

	0.0174***	0.0140***	0.0138***


***p-value < 0.01, OLS, ordinary least squares. ^1^Bivariate regression includes only TETRAS variable of interest with robust standard errors (Y = β_0_ + β_1_(TETRAS variable) + ɛ) ^2^Core model covariates also include age, employment status, household income, anxiety, and depression. (Y = β_0_ + β_1_(TETRAS variable) + β_2_(AGE) + β_3_(EMPLOYMENT_STATUS) + β_4_(ANXIETY) + β_5_(DEPRESSION) + ɛ). ^3^Expanded model covariates also include core model covariates plus sex, education level, and CCI. (Y = β_0_ + β_1_(TETRAS variable) + β_2_(AGE) + β_3_(EMPLOYMENT_STATUS) + β_4_(ANXIETY) + β_5_(DEPRESSION) + β_6_(SEX) + β_7_(EDUCATION_LEVEL) + β_8_(CCI) + ɛ).

### Work Impairment and Association with Tremor Severity

**Figure 2:**
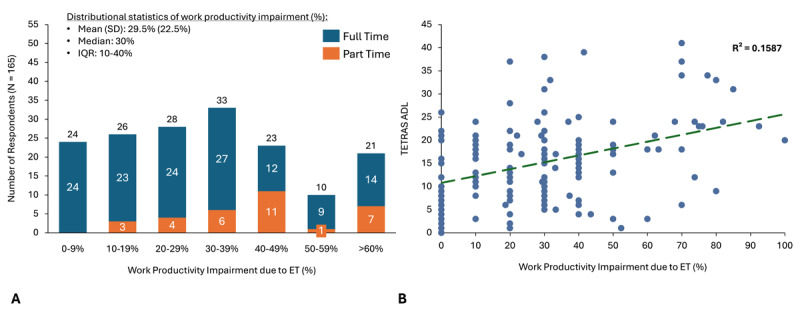
**Work productivity impairment by employment status in the Employed Subset of ET patients and association with tremor severity. A)** Work impairment as measured by the Work Productivity and Activity Impairment questionnaire, stratified by employment status (full-time, part-time, retired, unemployed, or student). **B)** Overall work impairment as measured on the Work Productivity and Activity Impairment questionnaire. Tremor severity as measured by TETRAS-ADL, R² = 0.1587.

**Figure 3:**
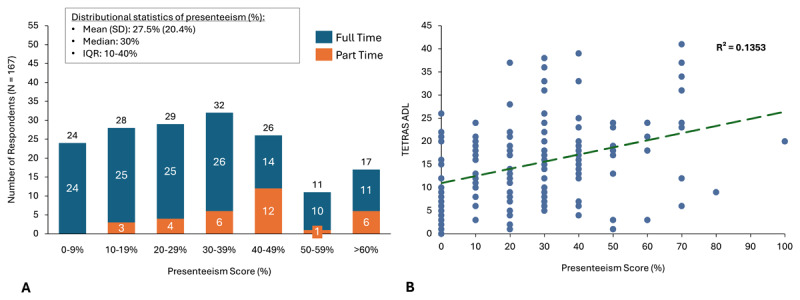
**Presenteeism stratified by full-time versus part-time employment status and association with tremor severity. A)** Number of respondents and degree of presenteeism measured by the Work Productivity and Activity Impairment questionnaire question 5, among full-time and part-time employed patients. **B)** Degree of presenteeism correlation analysis with TETRAS-ADL, R^2^ = 0.1353.

Most patients (133/165; 80.6%) in the Employed Subset were employed full-time and had some level of work impairment (141/165; 85.5%). Mean work productivity impairment (accounting for both absenteeism and presenteeism) among patients was 29.5% (SD = 22.5%), representing approximately 12 hours of work lost per 40-hour workweek (Figure 2A). Work productivity impairment was significantly associated with tremor severity as measured by TETRAS-ADL (Figure 2B). Among employed patients with ET reporting any work productivity impairment, a minority of patients report absenteeism (work time missed) due to their condition (mean, 4.4% (SD 13.7%) while most ET patients report some presenteeism (work productivity impairment) due to their condition, with mean of 27.5% (SD 20.4%) (Figure 3A), representing approximately 11 hours of work lost in a 40-hour workweek. Additionally, presenteeism was significantly associated with tremor severity as measured by TETRAS-ADL (Figure 3B). Over 90% of the work time lost was due to presenteeism versus absenteeism.

The QUEST provided more details to contextualize the impact of ET on working patients. Among those patients for whom QUEST responses were available, 47% (n = 64/135) of patients with ET working full-time and 88% (n = 36/41) of those working part-time reported that tremor was interfering with work. Three-fifths of the ET patients working part-time (61%, n = 19/31) indicated that they were doing so due to tremor.

**Figure 4:**
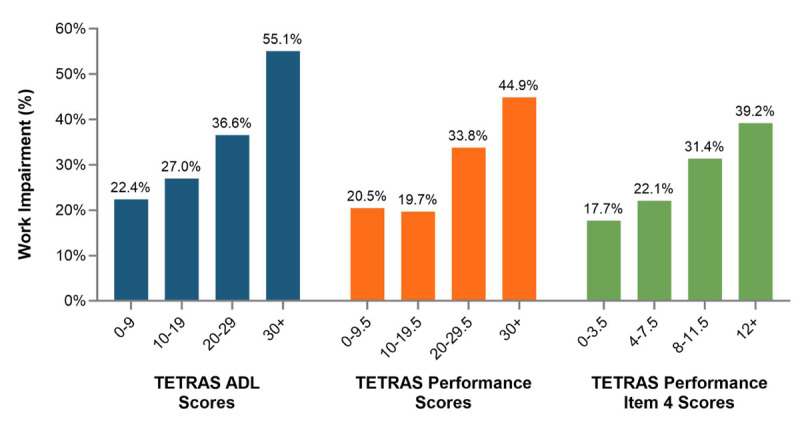
**Work impairment in association with tremor severity as measured by TETRAS in the Employed Subset of ET patients**. Scores are binned into four levels of tremor severity based on their TETRAS scores with corresponding mean percentages of overall work impairment as measured by the Work Productivity and Impairment Index.

Work productivity impairment observed in the Employed Subset was significantly associated with more severe (increasing) TETRAS-ADL, TETRAS-P, and TETRAS-P item 4 scores (p < 0.001 for trend; Figure 4). Higher patient work productivity impairment was moderately associated with greater tremor severity (rs = 0.32–0.44). For both presenteeism and absenteeism, associations between impairment and TETRAS subscales were moderate and statistically significant in regression modeling (Tables 3 and 4). The magnitude of relationships was not impacted by inclusion of covariates (age, full-time vs part-time, household income, depression, anxiety) in core models. Sensitivity analyses adjusting for sex, education level, and Charlson Comorbidity Index did not alter the associations (Tables 3 and 4).

**Table 3:** Presenteeism and association with tremor severity as measured by TETRAS subscales.


MODEL STRUCTURE	TETRAS ADL SCORE		

OLS model	Bivariate^1^	Core^2^	Expanded Model^3^

	0.0088***	0.0073***	0.0070***

	**TETRAS PERFORMANCE SCORE**		

OLS model	Bivariate^1^	Core^2^	Expanded Model^3^

	0.0078***	0.0071***	0.0071***

	**TETRAS PERFORMANCE ITEM 4 SCORE**		

OLS model	Bivariate^1^	Core^2^	Expanded Model^3^

	0.0159***	0.0130***	0.0130***


***p-value < 0.01, OLS, ordinary least squares. ^1^Bivariate regression includes only TETRAS variable of interest with robust standard errors (Y = β_0_ + β_1_(TETRAS variable) + ɛ) ^2^Core model covariates also include age, employment status, household income, anxiety, and depression (Y = β_0_ + β_1_(TETRAS variable) + β_2_(AGE) + β_3_(EMPLOYMENT_STATUS) + β_4_(ANXIETY) + β_5_(DEPRESSION) + ɛ). ^3^Expanded model covariates also include core model covariates plus sex, education level, and CCI (Y = β_0_ + β_1_(TETRAS variable) + β_2_(AGE) + β_3_(EMPLOYMENT_STATUS) + β_4_(ANXIETY) + β_5_(DEPRESSION) + β_6_(SEX) + β_7_(EDUCATION_LEVEL) + β_8_(CCI) + ɛ).

**Table 4:** Absenteeism and association with tremor severity as measured by TETRAS subscales.


MODEL STRUCTURE	TETRAS ADL SCORE		

Logit model	Bivariate^1^	Core^2^	Expanded Model^3^

	1.0677***	1.0742**	1.0736**

	**TETRAS PERFORMANCE SCORE**		

Logit model	Bivariate^1^	Core^2^	Expanded Model^3^

	1.0452**	1.0493**	1.0544**

	**TETRAS PERFORMANCE ITEM 4 SCORE**		

Logit model	Bivariate^1^	Core^2^	Expanded Model^3^

	1.0186	1.0127	1.0184


***p-value < 0.01; **p-value < 0.05; *p-value < 0.1; results presented in terms of odds ratios. ^1^Bivariate regression includes only TETRAS variable of interest with robust standard errors (Y = β_0_ + β_1_(TETRAS variable) + ɛ) ^2^Core model covariates also include age, employment status, household income, anxiety, and depression (Y = β_0_ + β_1_(TETRAS variable) + β_2_(AGE) + β_3_(EMPLOYMENT_STATUS) + β_4_(ANXIETY) + β_5_(DEPRESSION) + ɛ). ^3^Expanded model covariates also include core model covariates plus sex, education level, and CCI (Y = β_0_ + β_1_(TETRAS variable) + β_2_(AGE) + β_3_(EMPLOYMENT_STATUS) + β_4_(ANXIETY) + β_5_(DEPRESSION) + β_6_(SEX) + β_7_(EDUCATION_LEVEL) + β_8_(CCI) + ɛ).

## Discussion

This study showed that more than 4 out of 5 employed patients with ET have impairment in their daily activities and work. In this sample of employed ET patients, the degree of work productivity impairment was close to 30%, as assessed using the Work Productivity and Activity Impairment questionnaire. A large contribution of presenteeism to overall work impairment highlights the risk of significantly underestimating the true impact of ET on indirect disease burden if only absenteeism is considered. The association between activity and productivity impairment and tremor severity substantiates the impact of ET on patient functioning and emphasizes the wide-ranging burden of ET. Overall, the results of this study suggest that successful treatment of tremor severity should reduce the indirect burden of ET on patients and society.

These findings expand on the prior findings of tremor impact on work. Two prior studies examined the work/finance subdomains of the QUEST [4,9], with one group reporting a much higher percentage of patients reporting tremor interference with their job or profession than the other. Key differences between the two studies include the lower median age of patients (40.7 [9] vs. 69.7 years of age [4]) in the group reporting much greater tremor interference. However, neither study used a validated instrument to quantify degree of work productivity impact. Here, we estimate that close to one-third of the standard workweek may be lost to impaired work productivity in ET patients, chiefly in the form of presenteeism.

The study has several limitations. Given the sample recruitment method, the descriptive data may not be generalizable to all patients with ET. Patients with more severe ET or whose medical management is not optimized may make more frequent visits to their physician, and therefore, may be more likely to be included in the study population. While participants were required to have a physician diagnosis of ET, we acknowledge that these diagnoses were not required to follow formal clinical or research diagnostic criteria of ET. These analyses may not capture the full breadth of indirect impacts of ET, given the time horizon considered. Individuals who retired early or chose different career paths due to ET may not be represented in the work productivity impact reported here. Due to missing information for some patients on the QUEST, only partial data could be used to provide context to our findings.

In conclusion, work impairment is common among those ET patients who are employed, and this impairment is correlated with tremor severity. Future studies should incorporate work impairment data into estimates of indirect medical costs of ET.

## References

[1] Louis ED, McCreary M. How Common is Essential Tremor? Update on the Worldwide Prevalence of Essential Tremor. Tremor and Other Hyperkinetic Movements. 2021 Jul 9;11(1):28. DOI: 10.5334/tohm.632 34277141 PMC8269764

[2] Bhatia, KP, Bain, P, Bajaj, N, Elble, RJ, Hallett, M, Louis, ED, et al. Consensus Statement on the classification of tremors. from the task force on tremor of the International Parkinson and Movement Disorder Society: IPMDS Task Force on Tremor Consensus Statement. Mov Disord. 2018 Jan;33(1):75–87. DOI: 10.1002/mds.27121 29193359 PMC6530552

[3] Louis, ED, Collins, K, Rohl, B, Morgan, S, Robakis, D, Huey, ED, et al. Self-reported physical activity in essential tremor: Relationship with tremor, balance, and cognitive function. J Neurol Sci. 2016 Jul 15;366:240–5. DOI: 10.1016/j.jns.2016.05.034 27288815 PMC4936779

[4] Louis ED, Machado DG. Tremor-related quality of life: A comparison of essential tremor vs. Parkinson’s disease patients. Parkinsonism & Related Disorders. 2015 Jul 1;21(7):729–35. DOI: 10.1016/j.parkreldis.2015.04.019 25952960 PMC4764063

[5] Cersonsky, TEK, Diaz, DT, Kellner, S, Hickman, R, Zdrodowska, MA, Monin, JK, et al. Enfeeblement in Elders with Essential Tremor: Characterizing the Phenomenon and Its Role in Caregiver Burden. Tremor Other Hyperkinet Mov (N Y). 2019 Oct 18;9. DOI: 10.5334/tohm.461 PMC6814912 31709127

[6] Cersonsky, TEK, Kellner, S, Morgan, S, Cosentino, S, Koo, BB, de Figueiredo, JM, et al. Demoralization in essential tremor: prevalence, clinical correlates, and dissociation from tremor severity. CNS Spectr. 2020 Feb;25(1):16–23. DOI: 10.1017/S1092852918001633 30940264

[7] Huey, ED, Cosentino, S, Chapman, S, Azar, M, Rohl, B, Collins, K, et al. Self-report depressive symptoms are dissociated from tremor severity in essential tremor. Parkinsonism Relat Disord. 2018 May;50:87–93. DOI: 10.1016/j.parkreldis.2018.02.031 29499915 PMC5943134

[8] Busenbark KL, Nash J, Nash S, Hubble JP, Koller WC. Is essential tremor benign? Neurology. 1991 Dec;41(12):1982–1982. DOI: 10.1212/WNL.41.12.1982 1745359

[9] Chandran V, Pal PK. Quality of life and its determinants in essential tremor. Parkinsonism & Related Disorders. 2013 Jan;19(1):62–5. DOI: 10.1016/j.parkreldis.2012.06.011 22771281

[10] Furtado, J, Lally, C, Flanders, W, Gerbasi, M, Maserejian, N. Estimation of global age-specific prevalence of essential tremor by literature review of population-based studies [Internet]. MDS Abstracts; 2023 [cited 2025 Mar 27]. Available from: https://www.mdsabstracts.org/abstract/estimation-of-global-age-specific-prevalence-of-essential-tremor-by-literature-review-of-population-based-studies/.

[11] Saad, R, Markowitz, M, Gibbs, L, Fuller, D, Ni, W, Pahwa, R, et al. Diagnosed and Drug-Treated Prevalence of Essential Tremor in Adult Patients: Retrospective Analyses of Two US Healthcare Claims Databases (S51.004). Neurology. 2023 Apr 25;100(17_supplement_2):2450. DOI: 10.1212/WNL.0000000000202563

[12] Reilly MC, Zbrozek AS, Dukes EM. The Validity and Reproducibility of a Work Productivity and Activity Impairment Instrument. PharmacoEconomics. 1993 Nov 1;4(5):353–65. DOI: 10.2165/00019053-199304050-00006 10146874

[13] Anderson P, Benford M, Harris N, Karavali M, Piercy J. Real-world physician and patient behaviour across countries: Disease-Specific Programmes – a means to understand. Curr Med Res Opin. 2008 Nov;24(11):3063–72. DOI: 10.1185/03007990802457040 18826746

[14] Elble, RJ. The Essential Tremor Rating Assessment Scale. Journal of Neurology. 2016;5.

[15] Lyons K, Troster A, Tanner C, Pahwa R. QUEST: A disease specific measure of quality of life in essential tremor patients. In: Neurology. 2005. p. A254–5. DOI: 10.1037/t68731-000

[16] Quan, H, Li, B, Couris, CM, Fushimi, K, Graham, P, Hider, P, et al. Updating and validating the Charlson comorbidity index and score for risk adjustment in hospital discharge abstracts using data from 6 countries. Am J Epidemiol. 2011 Mar 15;173(6):676–82. DOI: 10.1093/aje/kwq433 21330339

